# Tobacco Smoke Exposure and Urinary Cadmium in Women from Northern Mexico

**DOI:** 10.3390/ijerph182312581

**Published:** 2021-11-29

**Authors:** Ángel Mérida-Ortega, Lizbeth López-Carrillo, Karla Rangel-Moreno, Natalia Ramirez, Stephen J. Rothenberg

**Affiliations:** 1 Centro de Investigación en Salud Poblacional, Instituto Nacional de Salud Pública, Cuernavaca 62100, Mexico; angel.merida@insp.edu.mx (Á.M.-O.); lizbeth@insp.mx (L.L.-C.); karlaambientales2012@gmail.com (K.R.-M.); nataliar12b@gmail.com (N.R.); 2 Rollins School of Public Health, Emory University, Atlanta, GA 30322, USA

**Keywords:** cadmium, tobacco, secondhand smoke, Mexico

## Abstract

Cadmium (Cd), a carcinogenic metal also related to reproductive and cardiovascular diseases, is contained in tobacco and elevated concentrations of it in humans have been consistently associated with first-hand tobacco smoke; however, there is scarce and inconclusive evidence of the relationship between Cd and secondhand smoke (SHS) exposure. Our aim was to evaluate the association between exposure to tobacco, both active and SHS, with urinary Cd concentrations in Mexican women. In a cross-sectional analysis that included 998 women living in northern Mexico, we measured the concentration of creatinine-adjusted urinary Cd (µg-cadmium/g-creatinine) using inductively coupled plasma triple quadrupole (ICP-QQQ) in tandem mass spectrometry mode (MS/MS). We gathered tobacco smoking information through an in-person interview and formed seven groups: non-smokers without SHS exposure; non-smokers with SHS exposure; ex-smokers without SHS exposure <1 year of quitting; ex-smokers without SHS exposure ≥1 year of quitting, ex-smokers with SHS exposure <1 year of quitting; ex-smokers with SHS exposure ≥1 year of quitting and current smokers. The interview also yielded sociodemographic characteristics. We used linear multivariable regression models to estimate the association between Cd concentrations and tobacco smoke exposure. Compared to non-smokers without SHS exposure, we found higher Cd concentrations in ex-smokers with SHS exposure <1 year of quitting and current smokers (adjusted geometric means 0.51 vs. 1.01 and 0.69 µg-cadmium/g-creatinine, respectively). Our results do not support a conclusion that SHS exposure is a source of Cd body burden.

## 1. Introduction

Cadmium (Cd) is a carcinogenic metal widely distributed in the environment that has also been associated with Itai-itai disease, kidney dysfunction, and bone damage, as well as alterations in the reproductive and cardiovascular systems [1,2,3]. Among the non-occupationally exposed population, diet and tobacco consumption are the main sources of exposure to this metal. Cadmium absorption after inhalation is greater than that through ingestion, so its concentrations can be four to five times higher among tobacco smokers compared to non-smokers [3,4,5]. Urinary Cd increases with age from the end of adolescence [6]. Blood Cd concentrations are lower in those with more education after adjusting for age [7]. It has been inconsistently related to breast cancer development [8]. In addition, urinary Cd concentrations among ex-smokers depended on time since quitting smoking [9,10]. Cadmium is an important toxic metal contained in tobacco. This metal enters the tobacco plant either by its absorption from the soil and/or by the application of fertilizers that contain it [11]. Due to the chemical similarity with zinc, Cd shares zinc transporters that distribute Cd in the tobacco plant [12]. Cadmium accumulates in tobacco leaves naturally [13] and is more efficiently transferred to tobacco smoke compared to other metals contained in cigarettes [11]. During active tobacco smoking, mainstream smoke from the tobacco column is inhaled from the mouth end of the cigarette, and between puffs secondary smoke is generated from the burning end of cigarettes [14]. Secondhand smoke (SHS), also called environmental tobacco smoke or involuntary or passive smoking [15], is a mixture of sidestream smoke and exhaled mainstream smoke, which contains more than 7000 harmful chemical compounds [14,15].

The literature has consistently reported a positive relationship between first-hand tobacco use and Cd concentrations [2,16,17,18,19]. However, there is scarce and inconclusive evidence of the relationship between Cd and SHS exposure. Investigators of a previous study of non-smokers found no association between SHS exposure and Cd concentrations in urine [18], while other studies reported a positive association in urine [14] and in blood [20]. Nevertheless, many of these studies, besides having poor confounder control, did not have an exposure category very likely to have no effect of smoking on participants’ Cd body burden, such as non-smokers without SHS exposure. The correlation between Cd in blood and urine has been reported to be 0.64 [21]. Non-smokers are not safe from breathing SHS despite opening windows, using air filters, or separating smokers from non-smokers in public spaces [20].

The Global Burden of Disease estimated that, in 2019 in Mexico, 57,000 tobacco-related deaths occurred, of which 10,458 were attributed to SHS [22]. Despite the markedly decreased smoking prevalence in Mexico during the first decade of the 21st century [23], prevalence of current smoking has remained stable (17.6%) during the last decade, regardless of government efforts to reduce exposure to tobacco such as tax increases, banning smoking ads on TV and radio, as well as increased public health announcements about health consequences of smoking. In addition, enforcement of smoke-free public spaces has been weak, which has resulted in small decreases in SHS exposure in government buildings, restaurants, and bars, while no changes have been observed in other public spaces [24]. Additionally, smoking in the home constitutes an important source of exposure to SHS that represents a challenge for smoking prevention and control programs. Countries such as the USA, Scotland, and Canada have made significant efforts to promote smoke-free homes resulting in an increase in households with complete smoking bans [25,26,27]. In Mexico, there are some messages warning of the danger of exposure to SHS at homes within the “México sin tabaco” campaign [28] and recognition of smoke-free homes within government programs [29]. 

In the current study, we evaluated the association between exposure to tobacco, both active and SHS, with urinary Cd concentrations in Mexican women.

## 2. Materials and Methods

This is a secondary analysis of the relationship between urinary Cd concentrations with active and SHS exposure in women who participated in a case–control study of breast cancer. The original study, performed during 2007 to 2011, evaluated environmental and genetic factors associated with breast cancer in northern states of Mexico [30].

We included 1045 histologically confirmed breast cancer incident cases and 1030 age-matched controls (±5 years). Detailed information regarding recruitment of the women is published elsewhere [30]. Briefly, cases were identified in the main public and academic hospitals; while controls were selected through the Master Sample Framework used for the National Health Surveys, which provides a probabilistic list of households in urban and rural areas. The inclusion criteria comprised a minimum age of 18 years and at least one year of residency in the study area, as well as no personal history of cancer among controls and no other type of tumor among breast cancer cases. The response rates were above 90%. The study followed the Declaration of Helsinki guidelines; and participants provided a written informed consent. The Ethics, Biosafety, and Research Committees of the National Institute of Public Health of Mexico approved the protocol.

Due to economic constraints, in this report we only measured Cd concentrations in cases with available breast cancer molecular subtype information (*n* = 499), as well as the same number of controls who were age-matched to cases.

### 2.1. Interviews

Trained personnel interviewed participants in person to collect information about their sociodemographic and lifestyle characteristics, including tobacco consumption. Interviews of controls were carried out at home, whereas breast cancer cases were interviewed at the hospital after diagnosis and before receiving any type of treatment (average time from diagnosis to interview was 2 months). We also measured height and weight to calculate body mass index (BMI = kg/m^2^). 

### 2.2. Tobacco

We considered women who had smoked less than 100 cigarettes in their lifetime as non-smokers, those with at least 100 cigarettes in their lifetime as smokers, and those who lived with at least one smoker at home and/or work as exposed to SHS. If a woman had not smoked at least 100 cigarettes in their lifetime, no more questions regarding active smoking were asked. Among smokers, we also asked if she currently smoked and, if yes, she was considered a current smoker; if not, she was classified as ex-smoker. We divided each group of ex-smokers into two groups, those with 1 year or more since quitting smoking and those with less than a year since quitting. Thus, seven exposure groups resulted: non-smokers without SHS exposure, non-smokers with SHS exposure, ex-smokers without SHS exposure <1 year of quitting, ex-smokers without SHS exposure ≥1 year of quitting, ex-smokers with SHS exposure <1 year of quitting, ex-smokers with SHS exposure ≥1 year of quitting, and current smokers. We excluded from this report 6 ex-smokers with no information regarding their quitting date.

### 2.3. Cadmium Determination 

A first morning void urine sample was usually collected on the same day as the interview. We collected samples in a sterile disposable polypropylene urine collection cup, and an aliquot of 4 mL of urine was prepared in a Cryovial and stored frozen at or below −20 °C.

The Lautenberg Environmental Health Sciences Laboratory of Icahn School of Medicine in Mount Sinai in New York City determined urinary Cd in a subsample of 998 women (499 cases and 499 controls). Analysts diluted samples (200 µL) with 8.8 mL of diluent solution that contained 0.5% nitric acid, 0.005% triton X-100, and mixed internal standard, in polypropylene trace metal free Falcon tubes (VWR^®^ Metal-Free Centrifuge Tubes). They also analyzed samples using matrix matched calibration standards using Agilent 8800 inductively coupled plasma triple quadrupole (ICP-QQQ) (Agilent technologies, Inc., Wilmington, DE, USA) in tandem mass spectrometry mode (MS/MS) [31]. They measured each sample five times and reported the mean of those replicates as the final concentration. To correct for the differences in sample introduction, ionization, and reaction rates in the reaction cell, they used internal standards (yttrium, indium, tellurium, and lutetium). The chemical analyst was blinded to the self-reported smoking group identity of each urine sample.

Quality assurance and quality control included analysis of initial and ongoing calibration verification standards [31]. The recovery percentage was 96%. Cadmium detection limit (DL) was 0.12 ng/mL; only 7.30% of samples were under it. According to the methodology previously described [32] in samples with Cd concentrations below the DL, we imputed their DL divided by two. The inter-day and intra-day coefficients of variation were 4.2% and 13.5%, respectively. Likewise, the coefficient of variation for nine internal duplicate samples was 9.5%. The laboratory performed a blind duplicate analysis of two samples obtaining a coefficient of variation of 12.6%.

The Department of Toxicology of the CINVESTAV-IPN determined creatinine to account for urine dilution by spectrophotometry using a commercial kit (Randox Creatinine Kit, Central de Diagnostica e Industria, CDMX, Mexico) with 1 mg/dL as detection limit (Randox, Antrim County, UK). 

**Figure 1:**
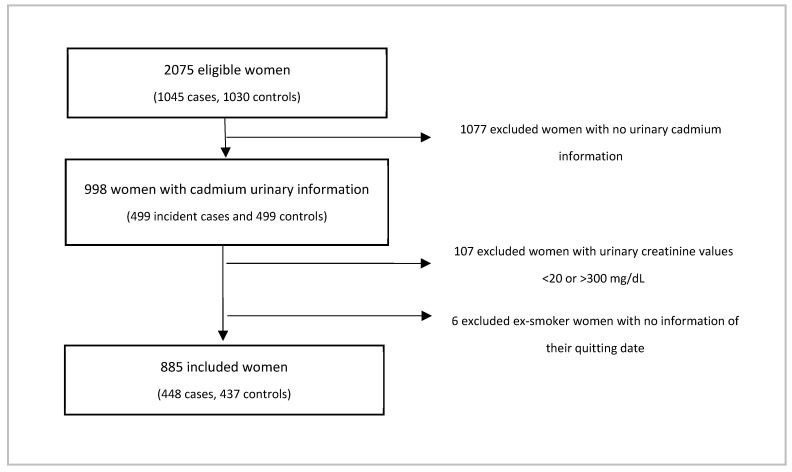
Selection of sample studied in this report.

We excluded participants with creatinine concentration <20 mg/dL or >300 mg/dL, as they may be related to clinical conditions that influence creatinine concentrations such as psychogenic polydipsia, creatine deficiency syndromes, muscular and renal disease, as well water adulteration in urinary samples [33,34,35]. In this way, the final sample in this report was 885 women (448 cases and 437 controls) (Figure 1). 

### 2.4. Statistical Analysis

Due to the small sample size, we grouped the neighboring states of Nuevo Leon (*n* = 233) and Tamaulipas (*n* = 7), as well as Coahuila (*n* = 152) and Durango (*n* = 47). We also compared selected characteristics among tobacco exposure groups through oneway ANOVA–Bartletts test or Kruskal–Wallis–Dunn test. In the case of residence state and breast cancer status, we presented percentages along with the results of multinomial logistic models comparing probabilities among smoking groups (dependent variable) by state of residence (independent variable) and by breast cancer status (independent variable), respectively. We selected the non-smoke without SHS exposure category as the base outcome and the Nuevo Leon/Tamaulipas state and women without cancer as the reference values, respectively. 

To correct for urine dilution, we divided Cd concentrations by creatinine [35]. In the total sample, we contrasted Cd concentrations among tobacco exposure groups; we also compared those concentrations among categories of characteristics of interest by each tobacco exposure group through an ANOVA test. We evaluated the association between natural log transformed Cd concentrations and tobacco exposure groups through linear regression models, and back-transformed natural log coefficients into geometric mean coefficients. The tobacco exposure groups variable was included as a categorical variable with a code for each group. We selected as covariates those that had Cd concentrations differences between any of the tobacco exposure groups with a *p*-value < 0.05: age, education, state of residence, and breast cancer status. We evaluated the relationship of continuous variables (age and education) with the outcome and included education as quartiles. In addition, we diagnosed the model graphically and with the Shapiro–Wilk test, assuming a normal distribution of the residuals, plotted the standardized residuals to assess heteroscedasticity and evaluated collinearity through the variance inflation factor. 

We performed the analyses using Stata 14 (StataCorp, College Station, TX, USA), and considered results as statistically significant at a *p*-value < 0.05.

## 3. Results

Among the tobacco exposure groups, ex-smokers without SHS exposure ≥1 year since quitting (mean = 61.5 years) were statistically older than other groups except ex-smokers without SHS exposure <1 year since quitting (mean = 47.5 years) and ex-smokers with SHS exposure ≥1 year since quitting (mean = 54.4 years), as well as being less educated than the rest of the women (mean = 4 years) (Table 1). We also observed tobacco consumption differences between states of residence similar to those reported by a national representative survey [36]. Compared to non-smokers without SHS exposure, we found a lower percentage of Nuevo Leon residents vs. those from Chihuahua (25.8% vs. 40.3, respectively) among ex-smokers with SHS exposure ≥1 year since quitting and current smokers (25.3% vs. 30.3%, respectively); as well as fewer women living in Sonora (33.1% vs. 19.6%, respectively) among non-smokers with SHS exposure. In addition, we observed that non-smokers with SHS exposure (66.5%) and ex-smokers with SHS exposure ≥1 year since quitting (71.0%) had a higher percentage of women with breast cancer, as well as a lower proportion of these women among ex-smokers without SHS exposure ≥1 year since quitting (38.3%) and current smokers (30.3%) (Table 1).

Compared with non-smoker women without SHS exposure, ex-smokers with SHS exposure <1 year since quitting and current smokers had higher urinary Cd concentrations (0.51 vs. 0.98 and 0.69 µg-cadmium/g-creatinine Geometric Means (GMs), respectively). Similarly, we observed some differences when comparing the concentrations of Cd among the categories of the variables of interest by each group of tobacco exposure. Among non-smokers without SHS exposure and current smokers, Cd concentrations were higher among older women compared to younger females (0.57 vs. 0.46 and 0.89 vs. 0.60 GMS, respectively); so too were they lower among women with more education versus less education (0.48 vs. 0.58 and 0.62 vs. 0.88 GMs, respectively). We also observed higher Cd concentrations in older women among ex-smokers with SHS exposure <1 year since quitting (2.23 vs. 0.76 GMs), as well as lower concentrations in those less educated ex-smokers with SHS exposure ≥1 year since quitting (0.54 vs. 0.87 GMs). In the group of ex-smokers with SHS exposure <1 year since quitting and current smokers, women from Chihuahua had higher Cd concentrations when compared to residents of Nuevo Leon/Tamaulipas (2.38 vs. 0.37 and 0.89 vs. 0.52 GMs, respectively). Among current smokers, women with breast cancer had lower concentrations of Cd compared to women free of this tumor (0.50 vs. 0.79 GMs) (Table 2).

In multivariate models, we detected higher Cd concentrations (µg-cadmium/g-creatinine) in ex-smokers with SHS exposure <1 year of quitting (adjusted Geometric Mean coefficient values (aGM) = 1.01 (95% CI 0.58, 1.45)) and current smokers (aGM = 0.69 (95% CI 0.58, 0.80)), compared with non-smokers without SHS exposure (aGM = 0.51 (95% CI 0.47, 0.55)), respectively (Table 3 and Figure S1). Complete model statistics are shown in Table S1. 

## 4. Discussion 

Our results showed that Cd concentrations in current smoking and in ex-smokers with SHS exposure with less than 1 year of quitting smoking are significantly higher than in non-smokers without SHS exposure (0.69 and 1.01 vs. 0.51 aGMs, respectively). 

Cadmium is widely distributed in the body, with the major portion of it found within the liver and kidney, with a half-life in urine between 14 to 24 years. The body burden of Cd reduces by 25% after one year of smoking cessation [9,13]. This could explain why ex-smokers <1 year of quitting smoking have higher urinary concentrations of Cd than non-smokers. Other researchers have previously studied the contribution of SHS exposure to the body burden of Cd, with inconclusive results [2,18,19]. Secondhand smoke was not related to urinary Cd concentrations, possibly due to the small sample size in a previous study [18] as well as in the present study. In contrast, there are other reports suggesting a positive association of SHS exposure with the Cd in the body [2,14,17,19,20,37]. 

Previous studies showed that current smokers have higher concentrations of Cd when compared with other groups, and that such relative magnitude depends on the reference group considered [2,17,18,19]. In this report, we estimated that current smokers had higher urinary Cd concentrations than non-smokers without SHS exposure (0.69 vs. 0.51 aGMs, respectively). In a 1999–2004 NHANES report, smokers had 38% higher urinary Cd concentrations than non-smokers with low SHS exposure. However, this report considered as non-smokers people that did not smoke in the 5 days prior to taking the sample [38], thus limiting our comparison. Research showed that urinary Cd concentrations of smokers are higher than those in non-smokers [2,18,19] and higher than in ex-smokers [2,17,18,19]. However, the aforementioned studies have only reported the mean urinary Cd concentrations for each group of smoke exposure, with a poor or no adjustment for possible confounders. In addition, the magnitude of the difference in Cd concentrations when comparing current smokers versus other groups also depends on the matrix used, and could be 4 to 5 times greater in blood [4,39,40].

Although the relatively small number of smokers and ex-smokers in our study may not have allowed us to detect SHS exposure effects on Cd concentrations among other groups, we note that the WHO states that SHS exposure disturbs the fundamental rights and freedoms of non-smokers [20]. To decrease SHS exposure in private spaces will require a combination of societal and individual action.

Our results must be interpreted considering some limitations. The contribution of SHS exposure to urinary Cd concentrations could depend on the number of smokers, as well as intensity and duration of smoking in current and ex-smokers which we did not consider in this report. The measure of duration of quitting smoking is also crude, limited to a binary division between those ex-smokers quitting in the same year of the interview and quitting in earlier years. Although the original study protocol specified that urine samples be collected on the day of the interview, there is uncertainty between the date of the interview and the urine sample collection as we do not have access to the date of sample collection. The use of questionnaires allowing the participants to self-classify themselves in the tobacco exposure groups does not allow ruling out possible bias that could place a current smoker into a lesser exposed group or a less exposed subject into a more exposed group. For instance, our question to identify current smokers “Do you currently smoke? (original Spanish: ¿Actualmente fuma?)” may also lead to uncertainty. In Spanish “actualmente” does not accurately define the duration of the behavior, e.g., not smoking at the time of the interview or some indefinite time before the interview. It is also possible that some recently started smokers had not yet smoked 100 cigarettes in their lifetime, yet were current smokers at the time of the interview. We do not know if this possibility was realized in the sample, as the protocol omitted the question “Do you currently smoke?” to those that had responded that they had not consumed at least 100 cigarettes in their lifetime. These types of misclassifications could serve to reduce the apparent difference in Cd between groups exposed to less smoke and the current smoking group. We do not account for other potential Cd exposures sources, such as house dust [41], occupation, or diet; however, we know that dietary contribution to urinary Cd is small compared to tobacco consumption [13]. Our small sample size may have reduced the power to detect an association between SHS exposure and Cd in most groups of our sample. However, the small difference in Cd between non-smokers with and without SHS exposure may not be large enough to be statistically significant. In addition, our results have limited application to the general population due to the study design used in the original study and the exclusion criteria that we added in this report. 

We also note some strengths. The chemical analyst was blinded to the self-reported smoking group identity of each urine sample and thus could not be a source of bias in the data. In addition, we were able to form an exposure category very likely to have had no effect on participant´s Cd body burden, i.e., non-smokers without SHS exposure, and also control for potential confounders in the studied relationship.

## 5. Conclusions

We have evidence that current smokers and ex-smokers with SHS exposure <1 year of quitting had higher Cd concentrations than non-smokers without SHS exposure. We have insufficient evidence to conclude that SHS exposure in our group of subjects is a significant contributor to body burden of Cd. The exclusion restrictions of the sample used in this report may limit broad application of the results to the general population.

## Figures and Tables

**Table 1:** Characteristics of study participants according to self-reported smoke exposure.

Characteristics	All (*n* = 885)	Non-Smoker without Secondhand Smoke Exposure (*n* = 402)	Non-Smoker with Secondhand Smoke Exposure (*n* = 245)	Ex-Smokers without Secondhand Smoke Exposure		Ex-Smokers with Secondhand Smoke Exposure		Current Smokers (*n* = 99)
				<1 Year of Quitting (*n* = 4)	≥1 year of quitting (*n* = 60)	<1 Year of Quitting (*n* = 13)	≥1 Year of Quitting (*n* = 62)	
Age, years [mean (SD)]	52.7 (12.4)	52.7 (12.4)	51.8 (12.5)	47.5 (16.4)	**61.5 (12.4) ^b^**	45.3 (10.5)	54.4 (11.2)	49.6 (10.5)
Education, years [median (P25, P75)]	6 (4, 9)	6 (3, 9)	6 (4, 10)	11.5 (4.5, 18)	**4 (1, 6) ^c^**	6 (5, 9)	6 (5, 12)	6 (5, 9)
Body Mass Index, kg/m^2^ [mean (SD)]	30.2 (6.0)	30.4 (6.3)	29.9 (5.9)	26.9 (6.3)	30.8 (5.9)	28.3 (6.0)	30.9 (4.8)	29.4 (5.9)
Creatinine, mg/dL [median (P25, P75)]	68.4 (41.0, 111.5)	64.7 (38.5, 111.5)	73.5 (44.0, 119.0)	55.7 (32.0, 147.0)	64.5 (39.2, 110.2)	67.9 (50.0, 102.4)	65.7 (49.0, 107.9)	71.0 (44.0, 104.5)
State of residence, [% (95% CI)]								
Nuevo Leon ^a^	27.1 (24.3, 30.2)	24.9 (20.9, 29.4)	**33.1 (27.4, 39.2)**	25.0 (0.5, 95.9)	23.3 (14.1, 36.0)	23.1 (6.3, 57.2)	**25.8 (16.2, 38.4)**	**25.3 (17.6, 34.9)**
Durango & Coahuila	22.5 (19.9, 25.4)	25.4 (21.3, 29.9)	23.3 (18.4, 29.0)	25.0 (0.5, 95.9)	13.3 (6.7, 24.9)	30.8 (10.2, 63.5)	14.5 (7.6, 26.0)	18.2 (11.7, 27.2)
Chihuahua	20.1 (17.6, 22.9)	11.7 (8.9, 15.2)	24.1 (19.1, 29.9)	25.0 (0.5, 95.9)	21.7 (12.8, 34.2)	23.1 (6.3, 57.2)	**40.3 (28.6, 53.2) ^d^**	**30.3 (21.9, 40.2) ^d^**
Sonora	30.3 (27.4, 33.4)	38.1 (33.4, 42.9)	**19.6 (15.1, 25.1) ^d^**	25.0 (0.5, 95.9)	41.7 (29.6, 54.8)	23.1 (6.3, 57.2)	19.4 (11.2, 31.5)	26.3 (18.4, 36.0)
Breast cancer cases, yes [% (95% CI)]	50.6 (47.3, 53.9)	42.5 (37.8, 47.5)	**66.5 (60.3, 72.2) ^d^**	100.0	**38.3 (26.7, 51.5) ^d^**	100.0	**71.0 (58.2, 81.1) ^d^**	**30.3 (21.9, 40.2) ^d^**

^a^ Includes 7 women from Tamaulipas; ^b^ Different from other groups except ex-smokers without secondhand smoke exposure <1 year since quitting and ex-smokers with secondhand smoke exposure ≥1 year since quitting; ^c^ Different from other groups; ^d^ Different from non-smoker without secondhand smoke exposure. Bold numbers correspond to statistically significant differences with a *p*-value < 0.05. Oneway ANOVA with Bartletts test [scheffe] for age and BMI; Dunn´s comparison [Kruskal-Wallis equality-of-populations rank test] for education and creatinine; Multinomial logistic models for state of residence and breast cancer status.

**Table 2:** Geometric mean urinary cadmium (µg/gr-creatinine) (95% CI) concentrations across characteristics of study participants according to self-reported smoke exposure.

Characteristics	Non-Smoker without Secondhand Smoke Exposure (*n* = 402)	Non-Smoker with Secondhand Smoke Exposure (*n* = 245)	Ex-Smokers without Secondhand Smoke Exposure		Ex-Smokers with Secondhand Smoke Exposure		Current Smokers (*n* = 99)
			<1 Year of Quitting (*n* = 4)	≥1 Year of Quitting (*n* = 60)	<1 Year of Quitting (*n* = 13)	≥1 Year of Quitting (*n* = 62)	
All	0.51 (0.47, 0.55)	0.54 (0.49, 0.59)	0.69 (0.20, 2.39)	0.58 (0.47, 0.71)	**0.98 (0.46, 2.09)**	0.61 (0.50, 0.76)	**0.69 (0.59, 0.80)**
Age, years							
22–52	**0.46 (0.41, 0.51)**	0.53 (0.47, 0.61)	0.59 (0.00, 114.13)	0.81 (0.53, 1.23)	**0.76 (0.30, 1.92)**	0.53 (0.39, 0.70)	**0.60 (0.50, 0.73)**
53–88	**0.57 (0.51, 0.63)**	0.54 (0.46, 0.64)	0.81 (0.00, 30922.05)	0.53 (0.42, 0.67)	**2.23 (0.34, 14.85)**	0.70 (0.51, 0.97)	**0.89 (0.69, 1.16)**
Education, years							
<6	**0.58 (0.52, 0.65)**	0.58 (0.47, 0.70)	1.87 ^b^	0.61 (0.47, 0.79)	0.76 (0.06, 12.57)	**0.87 (0.64, 1.17)**	**0.88 (0.66, 1.16)**
≥6	**0.48 (0.43, 0.52)**	0.52 (0.46, 0.59)	0.50 (0.14, 1.74)	0.54 (0.38, 0.75)	1.09 (0.48, 2.49)	**0.54 (0.41, 0.70)**	**0.62 (0.51, 0.74)**
Body Mass Index, kg/m^2^							
<25	0.50 (0.42, 0.59)	0.51 (0.40, 0.66)	0.59 (0.00, 114.13)	0.61 (0.26, 1.49)	1.48 (0.30, 7.37)	0.33 (0.20, 0.54)	0.57 (0.42, 0.79)
≥25 & <30	0.54 (0.49, 0.61)	0.56 (0.47, 0.66)	1.87 ^b^	0.51 (0.37, 0.71)	0.52 (0.10, 2.60)	0.67 (0.42, 1.07)	0.83 (0.63, 1.08)
≥30	0.49 (0.43, 0.55)	0.53 (0.46, 0.62)	0.36 ^b^	0.62 (0.48, 0.81)	1.16 (0.16,8.56)	0.65 (0.50,0.85)	0.65 (0.50,0.83)
State of residence							
Nuevo Leon ^a^	0.50 (0.43, 0.59)	0.52 (0.44, 0.62)	0.89 ^b^	0.61 (0.41, 0.91)	**0.37 (0.01, 13.08)**	0.47 (0.30, 0.75)	**0.52 (0.37, 0.73)**
Durango & Coahuila	0.54 (0.46, 0.63)	0.60 (0.48, 0.75)	0.39 ^b^	0.60 (0.27, 1.34)	1.04 (0.08, 12.82)	0.72 (0.35, 1.46)	0.72 (0.51, 1.02)
Chihuahua	0.51 (0.41, 0.63)	0.53 (0.42, 0.66)	0.36 ^b^	0.52 (0.28, 0.95)	**2.38 (0.27, 21.29) ^d^**	0.66 (0.45, 0.98)	**0.89 (0.67, 1.16) ^d^**
Sonora	0.50 (0.45, 0.55)	0.51 (0.42, 0.62)	1.87 ^b^	0.59 (0.45, 0.78)	0.97 (0.26, 3.57)	0.66 (0.48, 0.90)	0.64 (0.47, 0.89)
Breast cancer cases							
No	0.52 (0.47, 0.58)	0.61 (0.51, 0.74)	^c^	0.51 (0.39, 0.67)	^c^	0.49 (0.34, 0.70)	**0.79 (0.67, 0.93)**
Yes	0.49 (0.44, 0.55)	0.50 (0.45, 0.57)	0.69 (0.20, 2.39)	0.71 (0.51, 0.97)	0.98 (0.46, 2.09)	0.67 (0.52, 0.88)	**0.50 (0.36, 0.69)**

^a^ Includes 7 women from Tamaulipas; ^b^ Only one subject in this category; ^c^ No subjects in this category; ^d^ Different from Nuevo Leon. Bold numbers correspond to statistically significant differences within the variable with a *p* value <0.05. ANOVA tests for all variables.

**Table 3:** Regression analysis associations of adjusted geometric mean cadmium (µg/g-creatinine) (95% CI) among different self-reported smoking groups.

Model	Non-Smoker without Secondhand Smoke Exposure (*n* = 402)	Non-Smoker with Secondhand Smoke Exposure (*n* = 245)	Ex-Smokers without Secondhand Smoke Exposure		Ex-Smokers with Secondhand Smoke Exposure		Current Smokers (*n* = 99)
			<1 Year of Quitting (*n* = 4)	≥1 Year of Quitting (*n* = 60)	<1 Year of Quitting (*n* = 13)	≥ 1 Year of Quitting (*n* = 62)	
Model 1	0.51 (0.47, 0.55)	0.54 (0.49, 0.59)	0.71 (0.16, 1.26)	0.55 (0.44, 0.66)	**1.02 (0.58, 1.45) ^a^**	0.61 (0.49, 0.73)	**0.70 (0.59, 0.81) ^a^**
Model 2	0.51 (0.47, 0.55)	0.54 (0.49, 0.60)	0.74 (0.17, 1.31)	0.54 (0.43, 0.65)	**0.99 (0.57, 1.41) ^a^**	0.62 (0.50, 0.74)	**0.70 (0.59, 0.80) ^a^**
Model 3	0.51 (0.47, 0.55)	0.54 (0.49, 0.60)	0.76 (0.17, 1.34)	0.54 (0.43, 0.65)	**1.01 (0.58, 1.45) ^a^**	0.62 (0.50,0.75)	**0.69 (0.58, 0.80) ^a^**

Model 1 adjusted by age; model 2 additionally adjusted by state of residence and education; model 3 additionally adjusted by breast cancer status. GM (95% CI) of each covariable in model 3: age (mean) = 0.55 (0.52,0.58); state of residence = Nuevo Leon 0.52 (0.47, 0.57), Durango & Coahuila 0.58 (0.51, 0.64), Chihuahua 0.58 (0.51, 0.65), Sonora 0.54 (0.49, 0.59); education = 0–4 years 0.61 (0.54, 0.67), 5–6 years 0.54 (0.49,0.60), 7–9 years 0.58 (0.51, 0.65), 10–24 years 0.47 (0.41, 53); breast cancer status = no 0.56 (0.52, 0.61), yes 0.54 (0.50, 0.58). Bold numbers correspond to pairwise comparison, Tukey-corrected multiple comparison test with *p*-value <0.05, ^a^ compared to non-smoker without secondhand smoke exposure.

## Data Availability

Data sharing is not possible for ethical considerations.

## Supplementary Materials

The following are available online at https://www.mdpi.com/article/10.3390/ijerph182312581/s1, Table S1. Parameters of regression analysis between cadmium concentrations [ln(μg-cadmium/g-creatinine)] and self-reported smoking groups and Figure S1. Adjusted cadmium geometric means among different self-reported smoking groups from model 3.
