# Supplementary material for: Tobacco Smoke Exposure and Urinary Cadmium in Women from Northern Mexico
Source: Int J Environ Res Public Health. 2021 Nov 29;18(23):12581. doi: 10.3390/ijerph182312581 (PMC8656637; doi:10.3390/ijerph182312581)
Supplement: Supplementary file 1 [file ijerph-18-12581-s001.zip › ijerph-1418871-supplementary.pdf]

International Journal of
Environmental Research and Public Health

Supplementary Materials

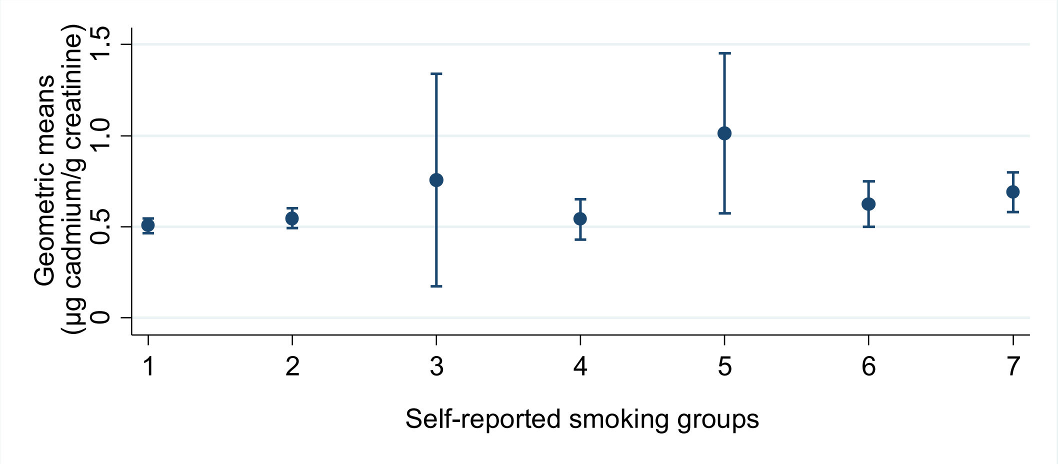

Figure S1. Adjusted cadmium geometric means among different self-reported smoking groups from model 3. 1 = Non-smoker without secondhand smoke exposure; 2 = Non-smoker with secondhand smoke exposure; 3 = Ex-smokers without secondhand smoke exposure <1 year of quitting; 4 = Ex-smokers without secondhand smoke exposure ≥1 year of quitting; 5 = Ex-smokers with secondhand smoke exposure <1 year of quitting; 6 = Ex-smokers with secondhand smoke exposure ≥1 year of quitting; 7 = Current smokers.

Table S1. Parameters of regression analysis between cadmium concentrations [ln(μg-cadmium/g-creatinine)] and self-reported smoking groups.

Model	F (Prob > F)	R-squared	Adjusted R-squared	Root-mean-square deviation
Model 1	3.81 (0.000)	0.030	0.022	0.787
Model 2	3.39 (0.000)	0.048	0.034	0.782
Model 3	3.18 (0.000)	0.049	0.033	0.783

Model 1 adjusted by age; model 2 additionally adjusted by state of residence and education; model 3 additionally adjusted by breast cancer status.
